# Assessing the Potential and Limitations of Leveraging Food Sovereignty to Improve Human Health

**DOI:** 10.3389/fpubh.2015.00263

**Published:** 2015-11-23

**Authors:** Andrew D. Jones, Lilly Fink Shapiro, Mark L. Wilson

**Affiliations:** ^1^Department of Nutritional Sciences, School of Public Health, University of Michigan, Ann Arbor, MI, USA; ^2^Department of Health Behavior and Health Education, School of Public Health, University of Michigan, Ann Arbor, MI, USA; ^3^Department of Epidemiology, School of Public Health, University of Michigan, Ann Arbor, MI, USA

**Keywords:** food sovereignty, public health, agriculture, nutrition, food systems

## Abstract

Food sovereignty has been defined as “the right of peoples to healthy and culturally appropriate food produced through ecologically sound and sustainable methods, and their right to define their own food and agriculture systems.” Human health is an implied component of this definition through the principle of healthy food. In fact, improved human health is commonly cited as a benefit of transforming food production away from the dominant practices of industrial agriculture. Yet, does the use of “ecologically sound and sustainable methods” of food production necessarily translate into better human health outcomes? Does greater choice in defining an agricultural or food system create gains in health and well-being? We elucidate the conceptual linkages between food sovereignty and human health, critically examine the empirical evidence supporting or refuting these linkages, and identify research gaps and key priorities for the food sovereignty-human health research agenda. Five domains of food sovereignty are discussed including: (1) use of agroecological management practices for food production, (2) the localization of food production and consumption, (3) promotion of social justice and equity, (4) valuation of traditional knowledge, and (5) the transformation of economic and political institutions and structures to support self-determination. We find that although there are many plausible linkages between food sovereignty and human health, the empirical evidence in support of the hypothesis that increasing food sovereignty yields improvements to human health is weak. We propose that a concerted effort to generate new empirical evidence on the health implications of these domains of food sovereignty is urgently needed, and suggest areas of research that may be crucial for addressing the gaps in the evidence base.

## Introduction

The concept of “food sovereignty” was first defined by the international peasant movement, *La Via Campesina*, in 1996 as “the right of peoples to healthy and culturally appropriate food produced through ecologically sound and sustainable methods, and their right to define their own food and agriculture systems” (1). In recent years, a global grassroots sociopolitical movement, coalescing around the principles of food sovereignty, has emerged and gained steady momentum. Hundreds of millions of indigenous peoples and small-scale peasant farmers have aligned with this movement (2), and at least two national governments, Ecuador and Bolivia, have outlined explicit policy frameworks centered on food sovereignty (3). In addition, a rich scholarly literature has emerged that explores the dynamics of this global movement, and its implications for participatory governance, social justice, industrial agricultural, and global trade (4–6).

Simultaneously, the public health agenda has been expanding beyond the examination of simple and direct causal links between particular exposures and disease-specific outcomes. New priorities within this agenda include studying social, behavioral, and environmental components that are linked to multidimensional impact pathways, and that aim to improve human well-being more broadly (7). In this context, human nutrition and health are often recognized as essential public goods (8) that are uniquely influenced by global systems of food and agriculture (9, 10). A resurgence of interest in the potential for food systems to shape public health has emerged in the past decade with the publication of numerous policy reports and academic articles, and the convening of several policy conferences addressing the topic (11–14). Yet, within the food sovereignty dialogue, the potential consequences of changes in food systems on human health and nutrition have received little attention. One recent review of nearly 1,500 articles addressing food security, food sovereignty, and health equity identified fewer than 20 reports involving food sovereignty (15). Indeed, food sovereignty narratives have predominantly centered on issues related to food production (e.g., land tenure, control of seeds, soil fertility management practices, and adaptive land management) (16–19) with less emphasis on the impacts of food systems on the wellbeing of consumers (15).

A recent edition of the *Nyéléni Newsletter*, a monthly letter centered on food sovereignty that is published jointly by *La Via Campesina* and many other organizations, explicitly addresses the topic of nutrition and food sovereignty, and highlights the growing attention that human health and nutrition are receiving from food sovereignty advocacy groups (20). Yet, most linkages between food sovereignty and health are considered complex and indirect, with a weak substantive base of empirical evidence to analyze these pathways. Accordingly, we undertook the present study to: (1) elucidate the conceptual linkages between food sovereignty and human health, (2) critically examine the empirical evidence supporting or refuting these linkages, and (3) identify research gaps and key priorities for the food sovereignty-human health research agenda.

## Defining Domains of Food Sovereignty

Since the genesis of the concept, authors have defined food sovereignty from multiple perspectives and positions. Much of the writing on food sovereignty has focused on large-scale, political and macro-economic issues such as agrarian reform, trade agreements, and land rights (21). Other dialogue on food sovereignty has emphasized access to local markets, application of agroecological farming methods, gender equity, community control of natural resources, seed sovereignty, as well as the rejection of industrial agriculture, international financial institutions, and the corporate food regime (6, 22–24).

In 2007, at a meeting of the “International Forum for Food Sovereignty” in Mali, participants defined six “pillars” of food sovereignty that encapsulate many of these elements. A seventh pillar was later added. The pillars state that food sovereignty: (1) focuses on food for people, (2) builds knowledge and skills, (3) works with nature, (4) values food providers, (5) localizes food systems, (6) puts control locally, and (7) allows that food is sacred (25). These pillars and the *La Via Campesina* definition of food sovereignty mentioned above provide an important framework for advocacy and research efforts related to food sovereignty. However, much like the definition of food security agreed upon at the World Food Summit in 1996, which emphasizes “physical and economic access to sufficient, safe and nutritious food to meet dietary needs and food preferences,” (26) these aspirational definitions contain broad language that does not always allow for clear operationalization of specific concepts for purposes of implementation or measurement. Indeed, one of the great strengths of the food sovereignty movement is the diversity of issues it addresses and its ability to respond to emergent challenges in different contexts such as climate change, control over access to natural resources, and local/global politics (27). However, measurement is critically important for identifying how and what to target, and with which policies to effectively and efficiently improve outcomes. Therefore, a clear conceptualization of food sovereignty is essential for understanding its potential implications for any number of outcomes, including human health and nutrition.

To more clearly define domains of food sovereignty that may be empirically measured and used to identify linkages between food sovereignty and human health, we propose five core domains of food sovereignty based on current definitions of the concept: (1) use of agroecological management practices for food production, (2) the localizing of food production and consumption, (3) promotion of social justice and equity, (4) valuation of traditional knowledge, and (5) transforming economic and political institutions and structures to support self-determination (Table 1). We describe each domain in depth below, its conceptual linkages to human health, and the evidence supporting these linkages.

**Table 1:** **Domains of food sovereignty and their conceptual linkages to human health**.

Domain	Description	Conceptual linkages to human health
Agroecological management	Use of agroecological practices for managing farm inputs and production	Differing nutritional quality of food
		Lower content of pesticides and toxic metals in food
		Positive effect of agrobiodiversity on diet diversity
		Direct exposure to pesticides and herbicides
		Exposure to zoonotic and water-associated vector-borne illness
Local production and consumption	Shift away from global trade and aggregation	Improved nutritional quality of food
	Emphasis on smallholder producers	Knowledge of/investment in food sourcing leads to positive dietary behavior changes
	Reliance on local community	Community engagement leads to increased well-being
	Food as more than commodity	Potential impacts on resilience, food security, and diet
	Rejection of “food dumping” as form of development assistance	
Social justice and equity	Equity in participation and sharing of benefits and risks of food systems	Distribution of health benefits to vulnerable groups
Traditional knowledge	Valuation of traditional knowledge, and culturally appropriate processes	Potential to reach isolated areas with efficacious approaches
	Rejection of technology or structures that undermine these	Platform for awareness building and education
		Loss of access to potential benefits of biotechnology
Self-determination	Capacity of individuals, organizations, and states to self-determination regarding resource management and production or consumption decisions	Restrictions on potential decisions or actions because of policy frameworks and economic structures that limit potential of other domains to impact health
		Trade-offs with potential to benefit or harm health depending on context and modifying factors

## Linkages Between Food Sovereignty and Human Health

Several conceptual frameworks have been proposed linking agriculture and food systems to human health and nutrition (10, 28, 29). These frameworks, each with unique components, highlight common pathways from agriculture to health and nutrition, including access to food for the producer’s own consumption, agricultural livelihoods and income from agriculture, impacts on labor allocation, control of resources, and gender relations, as well as the potential health effects of agriculture’s impact on natural environments. Similar pathways have been proposed for the linkages between food sovereignty and health equity, though social capital and cultural factors are more explicitly emphasized (15). These different frameworks have arisen in part as a response to persistent assumptions that achieving more economically efficient agricultural systems (i.e., systems that produce higher yields and greater profits) will translate into improved nutrition and health outcomes. Empirical evidence has consistently failed to support this assumption, and indeed, there is considerable evidence to suggest that nutrition and health goals must be explicitly incorporated into agriculture and food security projects if gains are to be made in improving these outcomes (30–32).

This evidence on the need for explicit nutrition and health goals is situated most closely within food security frameworks. The concept of food sovereignty may have different and new connections to human health as it extends beyond the traditional pillars of food security [i.e., availability, access, utilization, and stability (33)] to include principles of ecologically sustainable, locally defined and culturally appropriate food production and consumption, with a focus on people’s rights over global market concerns. However, given the complex determinants of human health and the limited evidence to date for the impact of agriculture and food systems on health, we hypothesize that increasing the food sovereignty of a particular individual, community or nation will not be sufficient in and of itself to improve health outcomes.

In the following discussion, we do not seek to reinvent or propose a new conceptual framework for the linkages between agriculture, food systems, or food sovereignty with human health. Rather, using the five domains of food sovereignty identified above and building from previous frameworks, we examine plausible pathways linking each domain to human health, assess the nature and strength of evidence supporting these pathways, and explore hypotheses for how greater food sovereignty may lead to improvements in human health. The identified domains are those previously emphasized in the food sovereignty narratives discussed above. The evidence supporting the linkages between these domains and human health was assessed through literature searches of the relevant empirical research related to each domain using keyword searches, comprehensive reviews of cited literature in identified studies, and through correspondence with expert colleagues. These searches were not systematic, nor were they intended to be. Rather, we identified seminal research studies and reviews that presented evidence related to conceptual pathways that we identified as critical and fundamental, and discuss this evidence in the context of the broader food sovereignty literature.

### Agroecological Management

Agroecology applies ecological concepts and principles to the management of agricultural systems and leverages the complex ecological processes inherent in agroecosystems (e.g., nutrient cycling, symbiosis, predator/prey interactions) to maximize productivity while minimizing external inputs and negative environmental impacts (34, 35). Promotion and widespread adoption of agroecological methods for food production is arguably one of the most consistent and conspicuous priorities of the food sovereignty movement (23, 36). Application of agroecological methods may provide numerous benefits to agroecosystems, such as improved resource use efficiency, pest regulation, and stability and provision of ecosystem services (37, 38). These management practices also have the potential to impact human health.

Among the many approaches to farming that employ agroecological management practices, organic agriculture is the most widespread and intensively studied (39). The empirical evidence from numerous literature reviews to date does not support the claim that the nutritional quality (i.e., the macro- and micronutrient contents) of organically grown foods differs from that of foods produced from synthetic pesticide-based or inorganic fertilizer-dependent, industrial agriculture (40–42). However, contamination of foods with pesticide residues and the toxic metal cadmium has been observed to be higher in foods produced from industrial agriculture as compared to organic methods (41, 42). Furthermore, concentrations of numerous polyphenolic compounds with antioxidant properties are higher in organic foods as compared to industrially produced food (41, 42). The meta-analyses that were conducted to review this evidence examined studies with highly heterogeneous methods, outcomes, and differing practical definitions of “organic agriculture,” making them difficult to interpret. Indeed, it is clear that we understand very little about the potential practical health implications of these observed differences in food produced using agroecological principles, making additional research necessary to disentangle these impacts.

In addition to the potential effects of farm management practices on the nutritional quality of foods, a growing body of literature suggests that farming households that cultivate a greater diversity of crops may benefit from more varied diets (43–46). Food diversity has long been recognized as the hallmark of healthy diets. Diets that include a diverse variety of foods are more likely to meet dietary requirements (47), and are associated with better anthropometric outcomes in adults and children (48), as well as improved health outcomes more broadly (49). Although the extent of market integration, dependence on subsistence crops, and control of household decision making are all important modifiers of these relationships, agroecologically managed systems that preserve or enhance agrobiodiversity have the potential to contribute to more diverse diets and therefore healthier people.

Use of agroecological methods may also prevent or introduce exposures to various occupational hazards and infectious diseases that impact human health. Direct and indirect exposure to pesticides and other agrochemicals applied to crops is responsible for a substantial burden of ill health (e.g., respiratory, gastrointestinal, reproductive disorders, and cancers), especially in developing countries (50–52). Ecological pest management obviates the need for pesticide inputs and would therefore provide protection to producers and consumers from harmful exposures to these agrochemicals. Agricultural laborers may also be exposed to harmful dusts, gases, and other airborne particulates that could adversely impact respiratory health (53). Many of these exposures may be present on both industrially and agroecologically managed farms, especially given that decomposition of organic materials may contribute to such exposures. Therefore, it is unclear how the potential health impacts of these exposures might be altered through application of different farm management practices. Finally, the transformative impact of agriculture on landscapes through changes in habitat, transport, and application of water for irrigation, and the introduction of livestock into managements systems, alters human exposure to water-associated, vector-borne illness and zoonotic diseases.

Although environmental disturbances should be less frequent and severe with agroecological than industrial management practices (54), any modification of human interactions with the environment likely poses some health risks. While agroecological methods will reduce synthetic toxin exposures, they may indirectly lead to increased contact with infectious microbes. These will be crop-, climate-, and context-dependent, but might involve water-associated infections (e.g., schistosomiasis, hookworm), vector-borne microbes (e.g., malaria, dengue, leishmaniasis), or zoonoses from exposure to livestock pathogens. Agroecological approaches, *per se*, will not necessarily reduce these microbial exposures, and might even increase some in the short term. Because agroecological methods are not a panacea for reducing human contact with disease-producing entities, a more informed and thoughtful integration of ecological food production and disease risk reduction is needed.

### Local Production and Consumption

Food sovereignty narratives implicitly or explicitly advocate for the localization of food systems in many different ways. Although these narratives are diverse and nuanced in their perspectives on international trade, they often promote national and regional food self-sufficiency, value smallholder farmers that produce for their own consumption or for local and regional markets, and emphasize a reliance on local communities for food sourcing (55). At the same time, advocates of food sovereignty are commonly critical of global trade and market integration through opposition to the liberalization of agricultural markets, the vertical integration of food supply chains, foreign direct investment in food processing and retailing, and the use of food aid development assistance to dispose of surplus production and expand markets (56). Taken together, such narratives emphasize local ownership and control of food systems that have profound implications for every link of food supply chains. However, the implicit health implications of localizing food systems are often presumed, but have not been rigorously analyzed.

There are several possible avenues via which localizing food systems may impact health. It is plausible that sourcing fruits and vegetables locally may improve the nutrient content of these foods. For example, fruits and vegetables that are sourced locally may: (1) spend less time in transit and therefore have less potential for nutrient loss, (2) have less handling and contact with machinery, and hence have less potential for damage and nutrient loss, (3) be minimally processed, thereby retaining beneficial nutrients and having fewer detrimental additives, or (4) represent varieties that were selected for nutritional quality rather than transportability (57, 58).

By strengthening community connections through the localization of food sourcing, in concert with promotion of healthy eating strategies, it may be easier to incentivize healthy dietary changes through enhanced self-efficacy and promoting awareness of food as a part of a healthy lifestyle. Indeed, it is clear that social connection in and of itself is a critically important determinant of overall health and well-being (59, 60). However, increased access to locally sourced food does not guarantee that people will make healthy dietary choices. In fact, the overall composition of diets may be more important for health than the particular nutrient content of specific foods (61).

Especially in low-income countries, poor households often face greater difficulties in achieving a diversified diet if food availability is limited to what is grown locally. These families, many of which are semi-subsistence farmers that consume a large share of what they produce, are acutely aware of where their food comes from. They may be limited to growing one or a few staple grains such as maize or rice, primarily for market, but also for their own consumption. Furthermore, the most nutrient-dense foods (e.g., fruits, vegetables, animal-source foods) may only be available for a short period of the year when procured locally. Therefore, strengthening external market linkages, not localizing the food system, is critically important both for improving the incomes of these farmers, and allowing them to diversify their diets.

At the same time, however, reliance on local resources may help to build resilience to outside shocks that could adversely impact food security. For example, when global rice prices rose to historically high levels in the spring of 2008, partly because of trade restrictions imposed by major suppliers, countries with rice reserves and diversified production strategies weathered the crisis better than those more dependent on rice imports (62). In addition, local, regional, or national food self-sufficiency in the face of climate-related natural disasters such as flood events and drought could also help to minimize adverse impacts on food security if food supplies and appropriate social safety net programs are able to quickly reach affected communities. In this sense, diversification of food production and procurement strategies at national, regional, and local levels, rather than isolationist, hyper-local solutions, may be most appropriate for building resilience to environmental or economic shocks. However, the scale of what constitutes “local” is critically important for understanding the context and potential health and nutrition implications of this aspect of food sovereignty.

### Social Justice and Equity

Ensuring equity of participation and the sharing of food system benefits and risks across class, race, and gender categories is another theme within food sovereignty narratives. The principles of social justice and equity are vitally important because of the linkages with human health, which are perhaps clearer than for any of the other domains of food sovereignty thus far identified.

As one example of the strong linkage between social equity and human health, an abundant literature documents the multiple, long-term health, nutrition, social benefits of girls’ access to primary education (63). Not surprisingly, promoting gender equality and empowering women have been a prominent fixture of the Millennium Development Goals of the United Nations for the past 15 years, and global development activities have incorporated such efforts into investments and programs across many sectors. Efforts to overcome barriers in access to resources, services, and information by marginalized ethnic, racial, and socioeconomic groups also consistently show health benefits across many different settings (64).

In the context of food systems, women farmers, especially those from poor households in low-income countries, are commonly among the most vulnerable to nutritional deficiencies and poor health outcomes. At the same time, these women often lack equal access to productive resources for agriculture such as land, labor-saving technologies, agronomic inputs, credit, extension, and support networks (65). Yet, evidence consistently shows that when women are able to access these resources, they are more productive, earn more income from their labor, and are able to gain time savings – one of their most precious resources (66). Furthermore, income controlled by women has a much greater positive effect on child nutrition and household food security than income controlled by men (67). In many contexts, as women’s status improves, so do health-seeking behaviors, appropriate complementary feeding practices for children, treatment of illness and immunization of children, and women’s and children’s nutritional status (68).

Therefore, to the extent that the food sovereignty movement is successful in promoting women’s access to and control of productive resources across the Global South, there should be substantial improvement in the health of marginalized women, children and other vulnerable groups. Also, it seems reasonable that as other priorities of the food sovereignty movement are advanced, synergistic effects on social equality are likely to appear. For example, revaluing traditional knowledge may help to empower women, as they are often the gatekeepers of such knowledge regarding food production, food preparation, and caregiving to safeguard health. Furthermore, greater equity in income sharing and decision making has been observed among households that use agroecological methods as compared to input-intensive agriculture (69). However, more evidence that addresses this association is needed (21).

The health benefits of reducing gender inequality and more broadly increasing social justice through changes in food systems must be more systematically scrutinized. Though gender equality is a priority in the food sovereignty movement (70), the principle of equity can often be used simply as a mobilizing ideology (71). Food sovereignty narratives must account for the diversity of experiences of women and other marginalized groups, and in doing so, articulate specific pathways of health impacts resulting from improved equity in different contexts. As an example, the assumption that women share common experiences and interests that lead them to adopt small-scale family farming practices, as compared to engagement with large-scale agribusiness, is not borne out by available evidence (71).

### Traditional Knowledge

One of the “pillars” agreed upon at the 2007 *International Forum for Food Sovereignty* in Nyéléni, Mali stated that food sovereignty “builds on the skills and local knowledge of food providers…developing appropriate research systems to support this and passing on this wisdom to future generations” (72). The valuation of traditional knowledge, then, is a key component of food sovereignty and one that may have implications for human health. There are many examples in public health of successful applications of traditional knowledge to cure disease and prevent illness. Use of oral rehydration salts (ORS) to treat childhood diarrhea is one such example. In Mexico, ORS began to be distributed as part of a national program in 1984, but supply fell far short of demand and poor access to vulnerable rural areas meant that those children who were most in need of treatment were not being reached. Health authorities, working with local communities and researchers, adapted traditional rehydration remedies based on medical science, and developed a culturally acceptable and efficacious treatment that overcame challenges of production, distribution, and cultural acceptability (73). There are many other examples of how traditional knowledge about soil, pest and water management has been applied to overcome agronomic challenges, while raising yields and preserving ecological functions (74). Similarly, there are other examples of traditional knowledge being leveraged to improve the nutritional quality of diets, prevent food-borne illness, and safeguard health (75). Traditional knowledge, therefore, has great potential to improve human health, especially in isolated regions where access to resources and services may be limited. In addition, traditional knowledge represents a platform for awareness building and broader education.

One of the peculiar challenges of applying traditional knowledge for nutrition-related illness is that malnutrition is often inconspicuous. In Latin America, for example, as in many other regions, a common phenotype for stunted children with a low height-for-age and high weight-for-age is “short and plump” (76, 77). Linear growth faltering in these children can easily go undetected in areas with a high prevalence of child stunting, as short height is seen as normal and children look otherwise “plump” and healthy. Therefore, complementing traditional knowledge with external perspectives and interventions can often be essential when faced with hidden challenges such as stunting.

Though traditional knowledge is valued within food sovereignty perspectives, scholars and advocates often define food sovereignty as much by the technology that the movement rejects as by the knowledge that it embraces. Genetic engineering is one such technology that is especially targeted, being seen to “undermine, threaten, or contaminate” traditional knowledge (72). Many of the challenges to genetic engineering within the food sovereignty movement have focused on genetically modified (GM) seeds and their potential to degrade the genetic diversity of seed stocks, disenfranchise farmers from control of their seed supply, and introduce financial hardship to poor farmers who must purchase new seed and inputs each season (78). The majority of commercialized GM crops that are currently available involve manipulations to increase tolerance to broad-spectrum herbicides or resistance to chewing insect pests, but increasingly there is emphasis on modifying other characteristics such as the climate resilience of crops (e.g., drought, salinity and high-temperature tolerance) (79). To the extent that the use of these seeds degrades seed genetic diversity in the long-term, or negatively impacts farmer livelihoods in the short-term (e.g., higher yields offset by higher prices paid for GM seeds), these outcomes could indirectly diminish household food security with concomitant adverse consequences for human health. However, the empirical evidence supporting claims of such negative consequences is limited. Several studies have in fact observed enhanced, short-term, production and economic potential among farmers planting GM seeds as compared to non-GM seeds (80, 81). Importantly though, there may be other indirect health effects of using GM seeds. Glyphosate, for example, the most widely applied herbicide worldwide (commonly known as Roundup^®^), used in concert with transgenic glyphosate-resistant crops, has recently been found to be “probably carcinogenic to humans” by the World Health Organization International Agency for Research on Cancer (82). Such health concerns warrant further research to elucidate the true potential for GM seeds to benefit or harm human health through these indirect pathways.

Regarding direct health effects of GM crops, many scientific bodies, governments, and organizations (e.g., American Association for the Advancement of Science, National Academy of Sciences, European Commission) have found that, based on currently available evidence, foods produced using transgenic or recombinant DNA technology pose no greater direct human health risks than foods produced using traditional plant breeding approaches (83). Available evidence, however, is incomplete, and additional research examining the three principal health concerns of GM crops (i.e., allergenicity, gene transfer, and outcrossing) is needed to continue to better understand potential direct health risks.

### Self-Determination

Perhaps the most obvious dimension of food sovereignty, yet the most difficult to circumscribe with clear boundaries, involves self-determination. Sovereignty and self-determination are concerned with independence and the freedom to choose. One strong area of emphasis within the food sovereignty movement has been deconstructing many of the structures related to globalization and neoliberal economic policy that are perceived to limit the independence and freedom to choose of specific stakeholders, especially poor farmers and consumers (84).

Policy shifts in the 1980s to liberalize agricultural markets in low-income countries, and later in the 1990s to open agri-food markets through trade agreements that reduced tariffs and export subsidies, underpin the recent sea change in global food systems. This change has brought about unprecedented shifts in how food is produced, transformed, transported, and accessed by consumers. Though many have argued that this transformation has largely fueled the precipitous increase in the global prevalence of obesity and other chronic diseases (85), many scholars present evidence to the contrary (86, 87). Taken as a whole, there is little evidence that these changes in agricultural markets have impacted health outcomes in a consistent manner. One of the primary reasons for this is that food and agriculture policies are commonly designed to incentivize or disincentivize production of specific crops. Yet, increasingly, crops produced through agriculture are not consumed directly, but are used as ingredients in processed foods. Therefore, the manner and extent to which these ingredients are substituted, transformed, and marketed in the postharvest food-value chain will largely determine their potential to shape diets and health outcomes (88). Indeed, food and agricultural policies affect both “healthy” and “unhealthy” foods and ingredients, and therefore, discerning a clear impact on health from these policies is not at all straightforward (88).

The legacy of colonial violence in many countries, and prevailing neoliberal socio-political and economic structures worldwide, continue to shape economic inequality, and the creation of dependency among nations. At the same time, globalization and the increasing integration of global markets has provided unprecedented access to information, resources, and economic opportunities that have lifted millions out of poverty. The simultaneous inequality and opportunity ushered in by this new global infrastructure is confounding, and the implications for global public health are uncertain. While hunger, undernutrition, and mortality from infectious illness have generally declined in the past quarter century, the prevalence of diet-related non-communicable disease has increased rapidly in all corners of the globe and is now the leading cause of death and disability in both developed and developing nations (89). Whether greater self-determination, or sovereignty, within food systems is a solution to halting the rise in chronic disease worldwide while ensuring continued gains in the prevention of undernutrition and infectious illness remains an open question.

## Toward a Food Sovereignty–Human Health Research Agenda

The domains of food sovereignty that we have identified surely do not encapsulate every aspirational component of food sovereignty as envisioned by the diverse peoples and organizations involved in this global movement. Rather, we have attempted to distill essential elements from this dynamic movement and from the body of scholarship on food sovereignty as they relate to human health. An understanding of these elements, in turn, may be used to guide investments in research to strengthen the empirical evidence base on the health implications of changes in food systems promoted by food sovereignty narratives.

Overall, the empirical evidence in support of the hypothesis that greater food sovereignty will yield improved human health is weak. This dearth of evidence may be due, in part, to the opposition that food sovereignty narratives pose to existing institutions, including health governance organizations (15). Furthermore, there are significant challenges of aligning food sovereignty related research with funding cycles and standard metrics of evaluation in health research (e.g., biomarkers) that commonly emphasize discrete solutions to clearly demarcated illnesses rather than a broader focus on systems dynamics that impact communities and populations (15).

Given this limited evidence, we propose that a concerted effort to generate new empirical evidence on the health implications of these domains of food sovereignty is urgently needed. We further propose that two areas of research in particular are crucial for addressing the gaps in the evidence base.

First, further research is needed to understand the specific pathways linking agroecology and human health. We argue here that decreased exposure to toxic chemicals and metals, both directly and through food consumption, as well as improvements in dietary diversity resulting from increased agrobiodiversity may represent boons for human health in conjunction with agroecological management practices. At the same time, exposure to infectious diseases associated with both agroecologically-­managed farms and industrial agricultural practices may pose adverse health risks. Yet, these pathways require further interrogation. In particular, research must elucidate the magnitude of importance of these pathways in different contexts. In addition, shifts toward agroecological management of farm systems can play a vital role in mitigating climate change (90). Climate change poses one of the greatest threats to human health and well-being given its scale and magnitude, and the multiplicity of health determinants that it affects (e.g., food and livelihood security, nutritional quality of foods, exposure to infectious microbes, natural disasters, and climate-related conflicts) (91). Greater evidence is needed, then, to understand how more climate-resilient agricultural systems, managed using agroecological approaches, may directly and indirectly impact human health. Furthermore, the purported impact of agroecology on more equitable social relationships, community cohesion, and the empowerment of women is a provocative hypothesis that should be further explored. This hypothesis is especially relevant given the increasing attention paid to social determinants of health that move beyond a narrow focus on the individual and encompass the health of communities and populations (92). If supported by evidence, these pathways could be among the most important for linking aspects of food sovereignty to human health.

Second, food sovereignty analyses that examine human health outcomes should place greater emphasis on the entire food supply chain. Agricultural production is only the most distal locus in an increasingly complex food supply chain that includes postharvest storage and home processing; industrial processing; distribution, transport and trade; food retailing, marketing and promotion; and food preparation and consumption (93). The increasing reliance on agriculture not as a source of food for direct consumption, but as source of inputs for the food processing industry (94), means that the raw commodities produced by agriculture will have a diminishing potential to directly impact human health as compared to the processes that reshape and transform these commodities postharvest. The emergence of ultra-processed foods (e.g., sugar-sweetened beverages, snacks foods, processed meats) has undoubtedly had far-reaching consequences for human health. The consumption of these foods has been linked to an increased risk of obesity, metabolic syndrome, and diet-related chronic disease (95, 96). Therefore, efforts by the food sovereignty community to restructure food supply chains may be even more effective at improving human health than efforts to reform agricultural production practices.

In addition, the principle of self-determination within the food sovereignty movement has just as much relevance to “downstream” components of food supply chains (e.g., food processing and marketing) as to “upstream” components related to agricultural production (e.g., control of seed systems and use of production inputs). For example, promoting local and regional cottage-based industries to process agricultural products could achieve multiple wins for low-income producers by enhancing income-earning opportunities, spurring multiplier effects in local rural economies, prolonging seasonal availability of nutrient-rich, perishable foods, and reducing food waste. All of these could plausibly benefit health through direct and indirect pathways. Similarly, safeguarding the content of media directed at children falls firmly within the mandate of reclaiming the sovereignty of food systems. Promotional marketing by food and beverage companies has enormous influence on consumer preferences, and has helped to transform diets globally. This is especially true of marketing to children, as they do not understand the persuasive intent of marketing and may develop long-term, unhealthy consumption habits that contribute to diet-related chronic disease, even before adulthood (97). Therefore, efforts to ensure that the marketing of foods and beverages, especially toward children, is aligned with goals of health promotion, is respectful of cultural values and norms, and is not controlled by profiteering external interests may be critical for achieving sovereign food systems. Research is needed to clarify the potential magnitude of effect of these efforts on health outcomes, and how these might differ across contexts.

## Conclusion

We identified five essential components of food sovereignty that provide a context for examining both the opportunities and limitations of how a food sovereignty framework may impact human health. We find that shifts toward more sovereign food systems have the potential to impact human health through both direct and indirect pathways that likely exhibit great heterogeneity across contexts. However, the evidence base supporting these pathways is limited. This warrants both caution regarding claims of the health benefits of changes to food systems that align with the goals of the food sovereignty movement, and also a new focus on generating the evidence needed to understand whether and how food sovereignty contributes to enhancing global public health and the level of individuals, families, and communities.

## Author Contributions

All authors contributed to the hypotheses put forth in the manuscript. LFS conducted an initial literature search to inform the findings of the manuscript. AJ wrote the first draft of the manuscript. All authors revised and approved the final manuscript.

## Conflict of Interest Statement

The authors declare that the research was conducted in the absence of any commercial or financial relationships that could be construed as a potential conflict of interest.
